# miRcorrNet: machine learning-based integration of miRNA and mRNA expression profiles, combined with feature grouping and ranking

**DOI:** 10.7717/peerj.11458

**Published:** 2021-05-19

**Authors:** Malik Yousef, Gokhan Goy, Ramkrishna Mitra, Christine M. Eischen, Amhar Jabeer, Burcu Bakir-Gungor

**Affiliations:** 1 Galilee Digital Health Research Center (GDH), Zefat Academic College, Zefat, Israel; 2 Department of Information Systems, Zefat Academic College, Zefat, Israel; 3 Department of Computer Engineering, Abdullah Gül University, Kayseri, Turkey; 4 Department of Cancer Biology, Sidney Kimmel Cancer Center, Thomas Jefferson University, Philadelphia, Pennsylvania, USA

**Keywords:** microRNA, Integrated, Machine learning, Grouping, Ranking, Gene expression

## Abstract

A better understanding of disease development and progression mechanisms at the molecular level is critical both for the diagnosis of a disease and for the development of therapeutic approaches. The advancements in high throughput technologies allowed to generate mRNA and microRNA (miRNA) expression profiles; and the integrative analysis of these profiles allowed to uncover the functional effects of RNA expression in complex diseases, such as cancer. Several researches attempt to integrate miRNA and mRNA expression profiles using statistical methods such as Pearson correlation, and then combine it with enrichment analysis. In this study, we developed a novel tool called miRcorrNet, which performs machine learning-based integration to analyze miRNA and mRNA gene expression profiles. miRcorrNet groups mRNAs based on their correlation to miRNA expression levels and hence it generates groups of target genes associated with each miRNA. Then, these groups are subject to a rank function for classification. We have evaluated our tool using miRNA and mRNA expression profiling data downloaded from The Cancer Genome Atlas (TCGA), and performed comparative evaluation with existing tools. In our experiments we show that miRcorrNet performs as good as other tools in terms of accuracy (reaching more than 95% AUC value). Additionally, miRcorrNet includes ranking steps to separate two classes, namely case and control, which is not available in other tools. We have also evaluated the performance of miRcorrNet using a completely independent dataset. Moreover, we conducted a comprehensive literature search to explore the biological functions of the identified miRNAs. We have validated our significantly identified miRNA groups against known databases, which yielded about 90% accuracy. Our results suggest that miRcorrNet is able to accurately prioritize pan-cancer regulating high-confidence miRNAs. miRcorrNet tool and all other supplementary files are available at https://github.com/malikyousef/miRcorrNet.

## Introduction

miRNAs are short non-coding RNAs of approximately 22 nucleotides and they have active role in controlling downstream proteomic profiles (Bartel, 2018). At the post-transcriptional level, miRNAs induce translational repression, mRNA deadenylation and mRNA decay via binding to their target mRNAs (Ivey & Srivastava, 2015). Hence, miRNAs are reported as one of the most important regulators of gene expression (Ivey & Srivastava, 2015). The potential role of miRNAs in regulating gene expression has opened the door to explore them as crucial therapeutic targets in complex diseases (Pencheva & Tavazoie, 2013).

It is predicted that approximately 30% of human genes (Lewis, Burge & Bartel, 2005) and nearly all cellular processes, including cell proliferation, apoptosis, necrosis, autophagy and stress responses, are regulated by miRNAs (Keller et al., 2011) (Ivanov, Liu & Bartsch, 2016). Since these processes are critical in carcinogenesis and tumor progression (Ling, Fabbri & Calin, 2013), miRNAs can be used as biomarkers for various cancer types, particularly to predict the likelihood of cancer development and progression.

Traditional analyses attempted to untangle the molecular mechanisms of carcinogenesis using a single -omic dataset, which contributed towards the identification of cancer-specific mutations, epigenetic alterations, etc. However, the acquisition of cancer hallmarks requires molecular alterations at multiple levels. The advancements in high-throughput technologies resulted in the production of mRNA and miRNA expression profiles for a sample at relatively lower costs. As the expression profiling has become routine experiment in biological laboratories, large expression data sets become available for several phenotypes. In this respect, the integrative analysis of -omics data, especially miRNA and mRNA expression profiling data could help to illuminate the above-mentioned regulatory mechanisms; to identify potential susceptibility pathways, diagnostic biomarkers, and to reveal novel and/or better therapeutic targets to treat cancer.

A review on miRNA-gene regulatory networks and their implications in cancer (Yousef, Trinh & Allmer, 2014) reported that miRNAs can form complex regulatory networks by themselves. Since miRNA expression is often tightly coordinated with gene expression, they form an intertwined regulatory network with many possible interactions among gene and miRNA regulatory pathways. This fact opens an interesting future work about integrated analysis of miRNA-expression with mRNA expression.

Integration of mRNA and miRNA expression profiles have been mainly performed using three different techniques, i.e. correlation, linear models, and Bayesian networks (Naifang, Minping & Minghua, 2013). Since miRNAs typically supress expression of their target genes, in correlation-based techniques, the correlation values between mRNA and miRNA pairs are calculated and hence, negatively correlated miRNA–mRNA pairs are chosen. However, correlation-based techniques assume that each miRNA affects a single mRNA. miRNAs often target more than one mRNA based on seed region sequence matches. Hence, one miRNA can affect more than one mRNA. In order to capture this relationship, a linear model is considered. Apart from these two techniques, Bayesian network-based approach is proposed. Using this probabilistic technique, mRNA–miRNA regulatory networks are generated. In addition to the correlation based, linear model and Bayesian network-based approaches; the following techniques have been proposed to discover miRNA–mRNA regulatory modules. Statistical approaches use statistical tests to find significant modules (Liu et al., 2010; Jayaswal et al., 2011; Yan et al., 2012; Hecker et al., 2013). Rule induction approaches use machine-learning methods to search for subgroups (Tran, Satou & Ho, 2008; Song et al., 2015; Paul et al., 2017). Probability-based approaches either use population-based probabilistic learning or probabilistic graphical model to infer regulatory information (Joung et al., 2007; Joung & Fei, 2009). Matrix decomposition approaches convert the integrated matrix derived from several types of information into several canonical forms (Zhang et al., 2011).

Most of the existing studies, as mentioned above, primarily detect miRNAs and mRNAs from differential expression (DE) analysis. Various correlation metrics are then used to determine the associations between these miRNA and mRNA pairs, which eventually construct mRNA–miRNA networks in specific cellular context. Besides these guilt-by-association based analyses, some studies constructed the networks from purely validated mRNA–miRNA association information, or in some cases they combined predicted associations to achieve better coverage; however, the latter may introduce higher false-positives (Huang, Morris & Frey, 2007; Long et al., 2013; Zhuang et al., 2015; Chou et al., 2018; Li et al., 2018; Liu et al., 2018; Yao et al., 2019; Yang et al., 2019).

As presented above, there are valuable studies that have integrated miRNA and mRNA expression profiles. But all these studies have some limitations. Firstly, most of these studies use various target gene prediction algorithms. As a result of the use of target gene prediction algorithms, the number of identified target genes can be up to 4,000. It is not feasible to validate such a huge number of target genes using low throughput methods such as luciferase reporter assays. Secondly, most of the existing studies (i) propose methods specific to a study, (ii) base their analyses on correlating mRNA and miRNA expression profiles, and (iii) intersect it with known databases (Peng et al., 2009; Gade et al., 2011). Although these studies provide insights, they do not provide software tools to reproduce the results or to use such a method for different diseases. Thirdly, almost all of these web-based and R-based tools are not updated frequently, and they are not easy to use for experimental biologists. Motivated by the limitation of these existing studies, in this paper we proposed a novel tool, miRcorrNet, which conducts machine learning-based integration of expression profiles. The tool integrates miRNA and mRNA expression profiles in order to detect miRNA-associated genes that are able to perform the classification task. The tool detects groups, which are later subject to the Rank procedure. The groups consist of a set of genes that are associated with a specific miRNA. The most distinctive feature of miRcorrNet is its ability to classify case and control samples (namely two classes) with an efficient performance using the acquired miRNA–mRNA groups. Thus, those groups of genes and their associated miRNAs may serve as a biomarker for the specific disease under investigation.

## Material & methods

### TCGA transcriptomic data analysis

We downloaded miRNA-seq and mRNA-seq expression profiles for 11 solid tumor types from The Cancer Genome Atlas (TCGA cancer) data portal (https://portal.gdc.cancer.gov/). For miRNA-seq profiles, raw read counts were normalized to reads per million mapped reads (RPM). For mRNA-seq profiles, the raw read counts were normalized to Reads Per Kilobase Million Mapped Reads (RPKM). In order to measure the correlations of miRNA–mRNA expression values, the Pearson correlation method was applied on the normalized expression profiles. To be able to separate cancer tissues from normal tissues, we used the standard cut-off value which is used in literature for RNA-seq data analysis (Mitra et al., 2020) For each cancer type, the miRNAs or mRNAs were selected for expression association analysis if at least 50% of the samples had a normalized expression value ≥1, as widely used in literature (Mitra et al., 2020).

For differential expression analysis of miRNAs and mRNAs, raw read counts were used as input into the R/Bioconductor package edgeR (Robinson, McCarthy & Smyth, 2010). The raw read counts were normalized with edgeR, based on the negative binomial distribution by using Trimmed Mean of M-values (TMM). We computed the differential expression of miRNAs and mRNAs in tumor samples compared with normal samples by estimating an exact test P-value, which is similar to Fisher’s exact test. The nominal P-values were adjusted by using the Benjamini–Hochberg (BH) multiple testing correction method.

### Our proposed method

**Figure 1:**
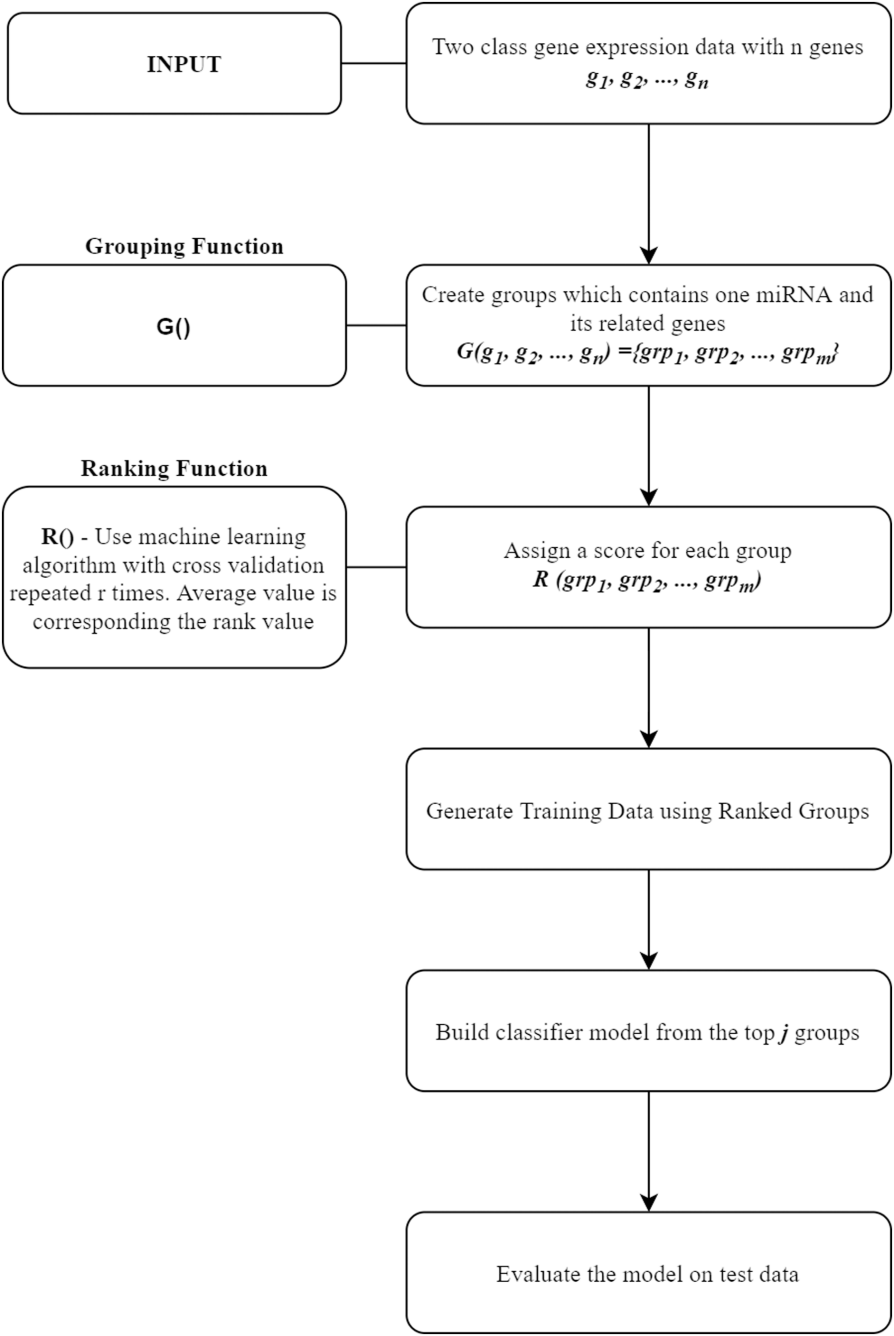
General workflow for classification based on grouping function G() and ranking those groups by R() fucntion.

In this section, miRcorrNet tool that we proposed in this study will be presented. miRcorrNet has been developed with an inspiration from our previously developed SVM-RCE, SVM-RNE, and maTE tools (Yousef et al., 2007; Yousef, Abdallah & Allmer, 2019; Yousef et al., 2009, 2020; Yousef, Ülgen, Uğur Sezerman, 2021). The general idea of these tools, demonstrating the main components is shown in Fig. 1. For a recent review, see Yousef, Kumar & Bakir-Gungor (2021). The general approach consists of two components, the Grouping function G() and the Ranking function R(). These two functions and their intended usage are illustrated in Fig. 1. Different methodologies can be used for the grouping operation. A computational grouping method such as K-means or another clustering algorithms as used in SVM-RCE (Yousef et al., 2007, 2020) are examples of this grouping function. Apart from that, biological information based grouping can also be applied (e.g., grouping based on target genes associated with a specific miRNA as in maTE tool (Yousef, Abdallah & Allmer, 2019)). Moreover, a hybrid grouping function can be defined using these two different grouping methods. The output of this G() function is a list of groups, where each group contains a set of genes. An example output of G() function for the Urothelial Bladder Carcinoma is shown in Table 1. In this table, miRNAs and their associated genes are shown. Table 1 actually presents the groups of miRNA that was created by calculating the correlations between mRNA and miRNA expressions. Each entry contains the list of genes that was correlated with the specific miRNA above the threshold (0.6). For example, hsa-miR-361-3p group contains SYNJ2BP, NLGN1, KCNK3 genes, as it can be seen in Table 1. The mRNA expression profiles of these genes are correlated with hsa-miR-361-3p profile; thus, we assume that those genes might be the targets of this specific miRNA. Now the next question is if we assume that our data only consists of these genes (SYNJ2BP, NLGN1, KCNK3) and a column of their class labels (a sub data of the original data), what is the significance of these genes in the task of separating the 2-class data. The answer of this question is given by the second component of our approach, which is the Ranking function R(), which assigns a score to each group (that is created by the G() function).

**Table 1:** Output of G() function applied on BLCA.

Name of the miRNA/group	Genes assocaited with the miRNA
hsa-miR-361-5p	CELF2, FBN1, LAMA4, NFIX, ENTPD1, AP1S2, ARHGAP24, HSPA12A, SYDE1, TSHZ3, NRP2, RAB3IL1, CCDC80, ABCD2, EMILIN1, MS4A2, SDC3, ROR2, ANGPTL2, STX2, SLC25A12, GAS7, LIX1L, SEC23A, SMOC2, ANXA6, ZEB2, ALDH2, GPR124
hsa-miR-361-3p	SYNJ2BP, NLGN1, KCNK3
hsa-miR-15b-3p	C9orf3, EMILIN1, CNRIP1, GPR124
hsa-miR-30e-5p	RAB3IL1, LIX1L
hsa-miR-181a-5p	SYNJ2BP, TACC2, JMY, ZDHHC15, MEIS1
hsa-let-7a-5p	FBN1, LAMA4, ENTPD1, SETBP1, EMILIN1, ANGPTL2, PDE3B
hsa-miR-22-3p	PDZRN3, NPR2, SCN7A, CSGALNACT1, GNAQ, SOX10, C5orf53, LAMB2, PJA2, NFIX, GRIK3, SPARCL1, AP1S2, TCEAL2, HECTD2, THRA, ADCY9, FAM149A, LOC653653, SYNE1, C4orf12, DCHS1, MS4A2, ABI3BP, PBX3, NR3C2, CNRIP1, UBE2Q2, RCAN2, PCDHGB7, RNASE4, ZDHHC15, RNF180, MYOT, SYT11, NAP1L2, STARD13, PLP1, GATA6, GRM7, TENC1, RAI2, SGCE, PLSCR4, GAS7, PKD2, TOR1AIP1, LIX1L, STAT5B, DCN, SMOC2, TCEAL7, LOC399959, RHOJ, ZEB2, ALDH2, PRIMA1, PCDH18, GPR124, KCNK3
hsa-miR-126-3p	LOC653653

The groups created in the G() step are used to create sub datasets from the original data, where each sub data consists of the genes that belong to a specific miRNA group keeping the original class labels. The Ranking function R(), as described in Table 2 is an approach that assigns a score for each group. In other words, after the ranking process, each group will have a score. This score expresses the ability of the relevant group to distinguish case and control classes. To assign a score, cross validation is used with a classification algorithm. The output of the Rank function R() is a list of group that are sorted by scores. Then, one can test the model on the top-ranked group or cumulatively on the top *j* groups. We choose *j* to be 10. In other words, we create sub data using the genes associated with the top ranked 10 groups, keeping the original labels. The model is created via applying the machine learning on this new sub data; and then the model is tested on the test set.

**Table 2:** Ranking algorithm for acquired miRNA–mRNA groups. The ranking method R() assigns a score for each group by performing an internal cross-validation.

**Ranking Algorithm -R(X**_**s**_**, M,*f,r*)** **X**_**s**_: any subset of the input gene expression data X, the features are gene expression values **M** { is a list of groups produced G() function} *f is a scalar*: split into train and test data r: repeated times (iteration) res={} for aggregation the scores for each *m*_*i*_ **Generate Score for each *m***_***i***_ ***and then rank according to the score, Rank(m***_***i***_***):*** For each *m*_*i*_ in M *sm*_*i*_=0; Perform *r* time (here r=5) steps 1-5: 1. Perform stratified random sampling to split X_s_ into train X_t_ and test X_v_ data sets according to *f* (here 80:20) 2. Remove all genes (features) from X_t_ and X_v_ which are not in the group *m*_*i*_ (Creat sub data that contains just the genes belongs to group *m*_*i*_ *)* 3. Train classifier on X_t_ using SVM 4. *t* = Test classifier on X_v_ –calculate performance 5. *sm*_*i*_ *= sm*_*i*_ *+ t;* *Score(m*_*i*_*)*= *sm*_*i*_ /*r* ; Aggregate performance *res*= { Union of Score(mi) } **Output** *Return {Rank(m*_*1*_*),Rank(m*_*2*_*),…,Rank(m*_*p*_*)} which is the sort of the list based on the score value of each group*

### miRcorrNet

Let us assume that we are given a two-class gene expression data *D*_*genes*_ for gene expression, and *D*_*miRNA*_ for miRNA expression over the same *N* samples. Table 3 is an example of the two input datasets. Let *L* and *K* be the number of genes in *D*_*genes*_ and *miRNA in D*_*miRNA*_, respectively. For simplicity, we use the terms gene and mRNA interchangeably.

**Table 3:** Example of input data for miRcorrNet.

mRNA expression data					
**Case ID**	**Class**	**A1BG**	**A2LD1**	**…**	**ZZZ3**
TCGA-DK-A6AV	neg	32.877	28.283	…	721.166
TCGA-DK-A3WX	neg	39.634	57.526	…	593.293
TCGA-GC-A3WC	pos	29.789	98.344	…	1,057.069
TCGA-BT-A20N	pos	37.378	55.011	…	755.688
…	…	…	…	…	…
**miRNA expression data**					
**Case ID**	**Class**	**hsa-let-7a-3p**	**hsa-miR-7a-5p**	**…**	**hsa-miR-99b-5p**
TCGA-DK-A6AV	neg	44.775623	13345.98449	…	9772.686386
TCGA-DK-A3WX	neg	34.30313	17531.35061	…	13508.08329
TCGA-GC-A3WC	pos	9.389	15229.41331	…	18601.78121
TCGA-BT-A20N	pos	3.534104	4717.325745	…	6845.372094
…	…	…	…	…	…

**Figure 2:**
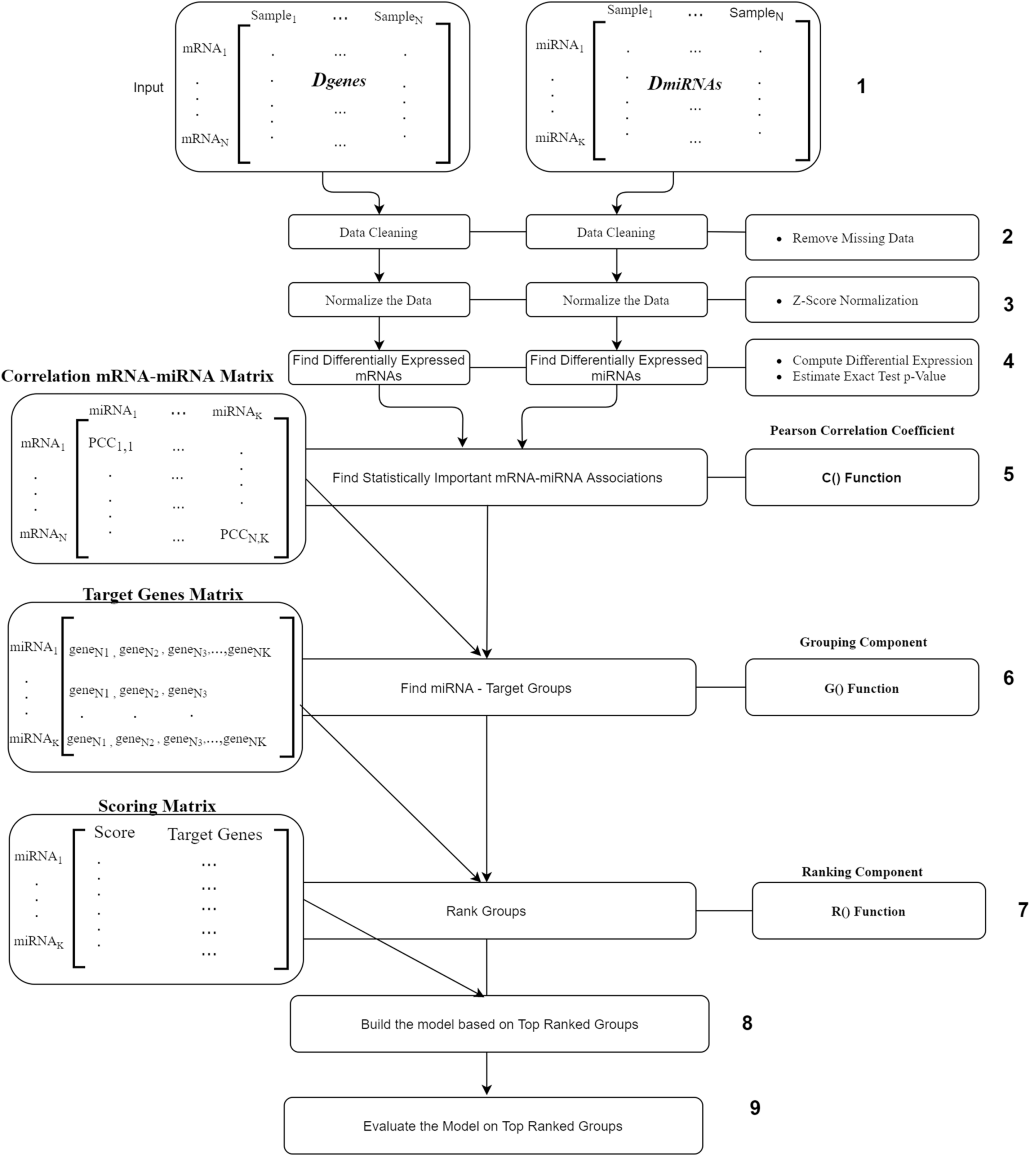
miRcorrNet Workflow.

The workflow of our suggested approach, miRcorrNet, is described in Fig. 2. The workflow consists of nine components where the most main components are the C(), G() and R() functions. The first component is the input component, where two data sets *D*_*genes*_ and *D*_*miRNA*_ are uploaded. The second and the third components are the removing of missing values and normalization, respectively. Next component is the fourth one where, differentially expressed genes and miRNAs are calculated using t-test. We only consider the mRNAs and miRNAs with p-values less and equal to 0.05. The fifth component is the C() function is the Pearson Correlation Coefficient which is to detect the mRNA–miRNA associations. This component uses the Pearson correlation coefficient in order to detect the set of genes that are negatively correlated with a specific miRNA. We set a stringent threshold as −0.6 in order to detect high-confidence associations. The output of C() is “miRNA-mRNA correlation” matrix that serve as input to the next component.

**Figure 3:**
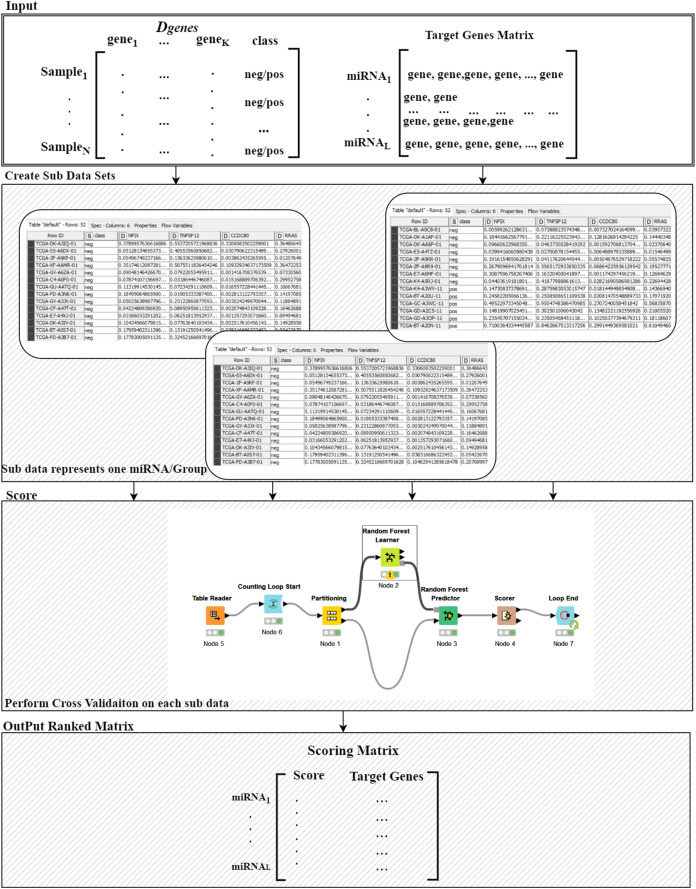
Details of the R() function.

The “Grouping Component” is the six component that actually integrates differentially expressed miRNAs and mRNAs in order to detect a group of miRNAs and its targets. In other words, this process corresponds to the G() function, which generates the groups that will be used to create sub data sets for each group, keeping the original class labels (as described in Fig. 3). This component creates the “Target Genes” matrix, which lists set of target genes for each miRNA. This matrix serves as an input to the seventh component named Ranking Component.

The Ranking component is applied to each gene group from the “Target Genes” matrix in order to estimate its significance in terms of separating the two-class data. The 8^th^ component is building the model based on top ranked groups. We have considered 10 models where the first one is built on the first ranked group while the second is built from the first two ranked groups and so on for the top ranked 10 groups. We mean by building on top groups is considering the genes that are associated on those groups.

The last component is the evaluation of the model created on top ranked groups.

For more details on the R() “Ranking Component” see Fig. 3. The input to this component is the original *D*_*genes*_ data and the “Target Genes Matrix”. For each miRNA entry of “Target Genes Matrix” we create a sub data of *D*_*genes*_ that contains just the genes listed in the miRNA entry keeping the “class column, as seen in “Create Sub Data Sets” component of the Fig. 3. This component creates L sub-data and each one of these datasets is used in the next component for performing scoring. The Score component loads the sub-data and performs cross validation procedure, recording the performance. We have used Random Forest classifier to build the model and perform the predictions. The output of this component is a Scoring matrix, which provides the score and the set of identified genes for each miRNA group. This Scoring matrix will be used to rank the groups and later to build a model using top j groups. Table 4 is an example of the output of the rank R() component. The model will be build considering the top groups genes.

**Table 4:** Example of the output of R() function applied on BLCA. Whole results for this R() output has been given as mean. The columns are the performance measurement achieved by cross-validation. The rows are the name of each group that is the miRNA. The sorted table according to Accuracy is used as the rank for each miRNA.

Group	Accuracy	Sensitivity	Specificity	Recall	Precision	F-measure	Cohen’s kappa
hsa-miR-32-5p	0.65	0.55	0.71	0.55	0.61	0.52	0.27
hsa-miR-361-3p	0.85	0.70	0.94	0.70	0.87	0.76	0.66
hsa-miR-205-5p	0.91	0.90	0.91	0.90	0.86	0.88	0.81
hsa-miR-30e-5p	0.76	0.60	0.86	0.60	0.77	0.60	0.46
hsa-miR-181a-5p	0.89	0.75	0.97	0.75	0.96	0.82	0.75
hsa-miR-106b-5p	0.93	0.85	0.97	0.85	0.96	0.88	0.83
hsa-let-7a-5p	0.78	0.65	0.86	0.65	0.75	0.68	0.52
hsa-miR-22-3p	0.95	1.00	0.91	1.00	0.89	0.94	0.89
hsa-miR-17-3p	0.91	0.80	0.97	0.80	0.96	0.85	0.79
hsa-miR-151a-5p	0.82	0.70	0.89	0.70	0.86	0.73	0.60
hsa-miR-374a-3p	0.69	0.55	0.77	0.55	0.57	0.55	0.32
hsa-miR-186-5p	0.84	0.75	0.89	0.75	0.83	0.78	0.65
hsa-miR-200c-3p	0.85	0.70	0.94	0.70	0.86	0.72	0.65
hsa-miR-576-5p	0.82	0.65	0.91	0.65	0.89	0.67	0.57
hsa-let-7a-3p	0.93	0.85	0.97	0.85	0.95	0.89	0.84

### Ranking the significance of miRNAs and genes in miRcorrNet

As seen in Fig. 2, miRcorrNet repeats the process N times. Each time 90% of the data is selected for training and the remaining 10% is selected for testing. Additionally, miRcorrNet randomly selects samples with a ratio of 1:2 for under-sampling. In each iteration our approach generates lists of miRNAs and their associated genes that are slightly different thus there is a need to apply a prioritization approach on those lists. Several genomic data analysis applications generate prioritized gene lists. Thus, we believe that the rank aggregation methods, as utilized in miRcorrNet, are useful solutions for the integration task. In this respect, we have embedded the RobustRankAggreg R package, developed by Kolde et al. (2012) into miRcorrNet. The RobustRankAggreg assigns a *P*-value to each element in the aggregated list, which describes how good each element/entity was ranked compared to the expected value.

In each iteration of N total iterations, as shown in Fig. 2, we rank the miRNAs according to the value given by R(). Then, we have N different lists with heterogeneous miRNA rankings. Those lists served as the input to RobustRankAggreg. Additionally, in order to rank the genes, we have assigned the rank of the miRNA for each gene. Then, we have N lists of ranked genes that also served to RobustRankAggreg for ranking.

### Implementation

The miRcorrNet is a next-generation solution based on the general approach described in Fig. 1, but it has been denationalised in terms of the mRNA–miRNA relationships. The miRcorrNet tool efficiently integrated mRNA and miRNA expression profiling data. The KNIME platform was used for the development of miRcorrNet tool (Berthold et al., 2008). This platform has been chosen since it is easy to use, and it is open-source software that can handle a wide range of operations. KNIME workflow consists of nodes with specific tasks. miRcorrNet is implemented as a KNIME workflow.

### Performance evaluation of the model

In order to evaluate model performance, for each established model, we calculated a number of statistical measures, such as sensitivity, specificity, and accuracy. The following formulations were used to calculate these statistics (with TP: true positive, FP: false positive, TN: true negative, and FN referring to false negative classifications):

Sensitivity (SE, Recall) = TP/(TP + FN)

Specificity (SP) = TN/(TN + FP)

Accuracy (ACC) = (TP + TN)/(TP + TN + FP + FN)

Additionally, we have calculated The Area Under the ROC Curve (AUC) measurements, which estimates the probability that a classifier will rank a randomly chosen positive instance higher than a randomly chosen negative instance.

All performance results reported in this study refer to the average of 100-fold Monte Carlo Cross-Validation (MCCV). MCCV is the process of randomly selecting (without replacement) some fraction of the data to form the training set, and then assigning the rest to the test set. This process is repeated multiple times, generating new training and test partitions each time randomly. We have chosen 90% for training and 10% for testing.

Some of the data sets used by the classifier are imbalanced. This situation can influence the classifier to the advantage of the data set with more samples; and it is well known as the problem of the imbalanced class distribution. We have applied an under-sampling approach, in which the number of samples of the majority class is reduced to the number of samples of the minority class. It reduces the bias in the size distribution of the data subsets. We have applied an under-sampling ratio of 1:2.

In our comparative evaluation experiments, we have tested miRcorrNet and maTE on the top 1 to top 10 groups, accumulatively. SVM-RFE is executed on different levels of genes, i.e., 1,000, 500, 250, 125, 100, 80, 60, 40, 20, 10, 8, 6, 4, 2, 1; and SVM-RCE is executed on the following clusters levels: 90, 72, 54, 35, 18, 13, 12, 11, 10, 9, 8, 7, 6, 5, 4, 3, 2, 1.

## Results

### Performance results

We have tested miRcorrNet on 11 high-quality cancer data sets, as listed in Table 5. Additionally, we have applied SVM-RFE (Guyon, Weston & Barnhill, 2002), maTE and SVM-RCE on those datasets. miRcorrNet uses both miRNA and mRNA expression profiles as an input, while the tools maTE and SVM-RFE consider just mRNA expression data. Table 6 presents a sample output of miRcorrNet, maTE and SVM-RCE. Table 7 presents an example of the output of miRcorrNet, based on the ranking of miRNAs, as determined from RobustRankAggreg method. In Table 8, we present the results obtained using the top two groups for maTE and miRcorrNet and the top 2 clusters for SVM-RCE. SVM-RFE does not have clusters or groups so we report the top 8 genes and top 125 genes.

**Table 5:** Used datasets details. Detail of the 11 datasets used to test miRcorrNet and other tools. Columns, normal and tumor are class labels while its value is the number of samples belonging to those classes.

TCGA cancer types	Abbreviation	Control	Case	Pubmed ID
Bladder urothelial carcinoma	BLCA	405	19	PMID: 24476821
Breast invasive carcinoma	BRCA	760	87	PMID: 31878981
Kidney chromophobe	KICH	66	25	PMID: 25155756
Kidney renal papillary cell carcinoma	KIRP	290	32	PMID: 28780132
Kidney renal clear cell carcinoma	KIRC	255	71	PMID: 23792563
Lung adenocarcinoma	LUAD	449	20	PMID: 25079552
Lung squamous cell carcinoma	LUSC	342	38	PMID: 22960745
Prostate adenocarcinoma	PRAD	493	52	PMID: 26544944
Stomach adenocarcinoma	STAD	370	35	PMID: 25079317
Papillary thyroid carcinoma	THCA	504	59	PMID: 25417114
Uterine corpus endometrial carcinoma	UCEC	174	23	PMID: 23636398

**Table 6:** Example of performance output of the tools based on eth general approach. This is an example of the output of the miRcorrNet, maTE, or SVM-RCE. This results acquired with miRcorrNet using BLCA data. The column #Genes is the average number of genes. In the first step. we build a model from the genes belonging to the first top group and then test it using the testing part of the data. Then we build a model from the top 1 and 2 groups then test. For j = 10. the model is built from the genes belonging to the top 10 groups and tested accordingly.

#Top groups	Number of genes	Accuracy	Sensitivity	Specificity
10	388.79	0.94	0.92	0.95
9	355.81	0.95	0.92	0.96
8	328.58	0.94	0.91	0.96
7	288.23	0.93	0.91	0.95
6	259.99	0.94	0.92	0.95
5	223.87	0.94	0.92	0.95
4	182.58	0.94	0.91	0.95
3	146.43	0.94	0.91	0.95
2	93.16	0.93	0.9	0.94
1	45.06	0.91	0.86	0.93

**Table 7:** Ranking miRNAs with RobustRankAggreg strategy using BLCA data. This table presents an example of the output of miRcorrNet based ranking of miRNA, determined from RobustRankAggreg method. Additionally, we have added genes in column three that are negatively correlated with the corresponding miRNA. The last column is the number of genes in each group associated with each miRNA.

miRNA	Score (*p*-value)	Targets	#Genes
hsa-miR-21-5p	8.42423E-33	RASGEF1C, SOX10, NOVA1, PCSK2, GRIK3, AR, EID1, ARHGAP6, C1QTNF7, CNTN2, TACC2, LYRM7, ZFP2, FAM149A, GPRASP2, FOXP1, TNNI3K, MID2, SYNE1, LRRTM1, RBM24, NR3C2, FAM54B, FOXF1, MEIS1, RNF180, MYOT, ZNF280D, SMAD9, PLP1, RAI2, NRXN1, CBX7, HERC1, MOAP1, LOC643763, MYST4, SERINC1, ZBTB4, PRIMA1, C20orf194	41
hsa-miR-22-3p	1.20936E-12	MEIS1	1
hsa-miR-16-5p	0.006982937	EVC, ZNF154, PPP3CB	3
hsa-miR-1976	0.011501451	FAM168B, AHNAK, ACOX2, PJA2, DNAJC18, F8, NFIX, ARHGAP24, TCEAL2, SETBP1, EVC, THRA, RNF38, ATL1, CRTC3, SETD7, GPRASP2, PLCL1, ZHX3, NFIA, DDR2, PBX3, KLHL13, ZFHX4, MEIS1, PBX1, RNF180, NFIC, KIAA1614, SLC24A3, EPDR1, HERC1, TOR1AIP1, SERINC1, NEK9, ZEB2, GPR124	37
hsa-miR-182-5p	0.125595903	CSGALNACT1, ACOX2, CD99L2, ARHGAP24, LRRK2, ROR2, ZEB2, ALDH2	8
hsa-miR-576-5p	0.126381607	MID2, ZBTB4	2
hsa-miR-92a-3p	0.301933719	SOX10, NOVA1, AQP1, EVC, LRRK2, MID2, ARHGAP1, C10orf72, SLC24A3, ALDH2	10
hsa-miR-26b-3p	0.92385325	SOX10, RRAGD, ARHGAP24	3

**Table 8:** Comparison results using all 11 datasets. Column *AUC* is Area Under the Curve. All the values are averaged over 100 MCVV for the level top 2 groups for maTE and miRcorrNet, while 8 and 125 genes for SVM-RFE and finally for SVM-RCE an average of 190.05 genes from cluster level 2. Standard deviation values is given for AUC.

Method	Number of genes	Accuracy	Sensitivity	Specificity	AUC	Standard deviation
miRcorrNet	141.1	0.96	0.94	0.97	0.98	0.05 ± 0.05
maTE	7.48	0.96	0.94	0.96	0.98	0.034 ± 0.026
SVM-RCE	190.05	0.96	0.94	0.97	0.99	0.06 ± 0.03
SVM-RFE	8	0.84	0.85	0.85	0.91	0.07 ± 0.04
SVM-RFE	125	0.96	0.97	0.95	0.98	0.05 ± 0.03

In general, there were no significant differences between the results of the 4 tools presented in Table 8. However, our aim for miRcorrNet is not to improve the performance of an existing tool. Rather, miRcorrNet intends to provide a deep analysis for experimental biologists and clinicians. In terms of deep analysis, miRcorrNet offers a list of mRNAs and miRNAs that are found to be potentially important for the disease under study. miRcorrNet lists individual mRNAs and miRNAs according to both their ranking results and their frequencies. In addition, miRcorrNet lists mRNAs that are targets of miRNAs. These relationships are ranked according to the p-values in ascending order, and it reveals the relationships acquired from the data. Furthermore, to separate the two classes, namely case and control, miRcorrNet runs the model it creates for each group, and makes a record of these results in the output file. Moreover, the results show that miRcorrNet performs as well as other tools in terms of accuracy. The results of miRcorrNet on all test sets are presented in Table 9. We have shown the performance results for the top 1, 2, 5, 7 and 10 groups. While the upper table shows the performance results, the lower table displays the number of genes (on average) corresponding to the top groups used in the upper table. In all tested datasets, the number of genes is low (The range is [8,69] for #Grp1), except for KICH data with 306 genes for #Grp 1. This performance results indicate that using only one top group (genes in the top ranked group), one can get very high accuracy in terms of distinguishing cases from controls. It means that the set of genes in the top ranked group is a good signature of the disease and could be used as a biomarker for the disease under study. For all other results, see Supplementary File located at https://github.com/malikyousef/miRcorrNet.

**Table 9:** miRcorrNet results. Whole miRcorrNet results has shown using Area Under the Curve (AUC) value in terms of performance. #Grp is the number of top groups. Number of genes mean values has been given.

	miRcorrNet performance										
**#Grp**	**BLCA**	**BRCA**	**KICH**	**KIRC**	**KIRP**	**LUAD**	**LUSC**	**PRAD**	**STAD**	**THCA**	**UCEC**
**10**	0.98	1.00	1.00	0.99	1.00	1.00	1.00	0.95	0.96	1.00	0.99
**7**	0.98	1.00	1.00	0.99	1.00	1.00	1.00	0.95	0.98	1.00	0.99
**5**	0.99	1.00	1.00	0.99	1.00	1.00	1.00	0.96	0.97	1.00	0.99
**2**	0.97	1.00	1.00	0.99	1.00	1.00	1.00	0.96	0.98	1.00	0.99
**1**	0.97	0.99	1.00	0.99	1.00	1.00	1.00	0.95	0.93	0.99	0.99
	**miRcorrNet number of genes**										
**#Grp**	**BLCA**	**BRCA**	**KICH**	**KIRC**	**KIRP**	**LUAD**	**LUSC**	**PRAD**	**STAD**	**THCA**	**UCEC**
**10**	407	56	4,916	245	365	352	398	122	86	278	389
**7**	290	60	2,998	207	316	257	270	69	52	219	269
**5**	211	49	2,031	162	297	181	194	54	26	173	193
**2**	84	32	870	70	157	65	68	21	13	92	75
**1**	46	24	306	35	69	29	28	10	8	48	33

miRcorrNet generates three output files. The first file lists the name of the miRNA and its significance with additional information. The second file is the list of genes, sorted by significance, and the third file includes the performance results.

### Validation of miRcorrNet’s findings on miRNA-disease association databases

miRcorrNet generates a file showing the association of diseases and miRNAs. The outputs in this file are produced using the ranking strategy. Each miRNA is ranked in descending order of score values indicating its association with each disease. The reliability of miRNA-disease associations, which we think is related to the disease, needs to be proven. For this purpose, the findings of miRcorrNet were compared with database entries, which keep miRNA-disease associations. For this purpose, we used dbDEMC (Yang et al., 2010), miR2Disease (Jiang et al., 2009), miRCancer (Xie et al., 2013) and HMDD (Huang et al., 2019). In order to limit the number of identified miRNAs for comparison, we set the association score threshold as 1. This threshold corresponds to the top 7 miRNAs as minimum number and the top 22 miRNAs as the maximum number among all tested disease datasets. We found that at least 90.47% and at most 100% of the relationships that are identified by miRcorrNet exists in the above-mentioned databases. A summary of this comparison is shown in Table 10. This table shows the miRNA-disease associations (obtained from databases) and the scores found by miRcorrNet. In the evidence column, the source of the disease-miRNA association is shown. Additionally, miRcorrNet found additional miRNAs, ‘hsa-let-7g-5p’ as associated with BLCA and ‘hsa-miR-1301’ as associated with UCEC.

**Table 10:** Comparison of miRNA-disease associations between miRcorrNet findings and existing associations in databases.

miRNA name	Score	Evidence	miRNA name	Score	Evidence
**BLCA**			**BRCA**		
hsa-miR-21-5p	7.32	dbDEMC, miR2Disease, miRCancer	hsa-miR-21-5p	9.66	dbDEMC, miR2Disease, miRCancer
hsa-miR-22-3p	4.67	miRCancer	hsa-miR-10b-5p	7.98	dbDEMC, miR2Disease, miRCancer
hsa-miR-148b-3p	4.06	dbDEMC, miR2Disease	hsa-miR-200c-3p	5.26	dbDEMC, miR2Disease, miRCancer
hsa-let-7g-5p	–	No evidence	–	–	–
**KICH**			**KIRP**		
hsa-miR-222-3p	9.33	dbDEMC	hsa-miR-21-5p	8.62	dbDEMC, miR2Disease, miRCancer
hsa-miR-221-3p	8.1	dbDEMC, miR2Disease	hsa-miR-10b-5p	4.95	dbDEMC, miR2Disease, miRCancer
hsa-miR-96-5p	7.03	dbDEMC	hsa-miR-589-5p	4.27	dbDEMC
**KIRC**			**UCEC**		
hsa-miR-28-3p	7.96	dbDEMC, miR2Disease	hsa-miR-151a-5p	2.23	dbDEMC
hsa-miR-21-5p	6.35	dbDEMC, miR2Disease, miRCancer	hsa-miR-200b-3p	2.12	dbDEMC, miRCancer
hsa-miR-106b-3p	6.17	dbDEMC, miR2Disease	hsa-miR-141-3p	2.01	dbDEMC, miRCancer
–	–	–	hsa-miR-1301	–	No evidence
**LUAD**			**LUSC**		
hsa-miR-30a-3p	6.23	dbDEMC, miR2Disease, miRCancer, HMDD	hsa-miR-146b-3p	3.76	dbDEMC, miR2Disease
hsa-let-7a-5p	6.13	dbDEMC, miR2Disease	hsa-miR-181a-5p	3.74	dbDEMC
hsa-miR-22-3p	5.49	dbDEMC, miR2Disease,	hsa-miR-205-5p	3.44	dbDEMC, miR2Disease
**PRAD**			**STAD**		
hsa-miR-143-3p	3.31	dbDEMC, miR2Disease	hsa-miR-21-5p	9.59	dbDEMC, miR2Disease, miRCancer
hsa-miR-375	3.04	dbDEMC, miR2Disease	hsa-miR-148b-3p	3.22	miRCancer
hsa-miR-200c-3p	1	dbDEMC	hsa-miR-185-5p	2.39	dbDEMC, miRCancer
**THCA**					
hsa-miR-152	7.26	dbDEMC			
hsa-miR-30a-5p	6.56	dbDEMC, miRCancer			
hsa-miR-148b-3p	6.5	dbDEMC			

### Validation on external data

We have evaluated the performance of miRcorrNet using an external dataset GSE40419, downloaded from the Gene Expression Omnibus database (Barrett & Edgar, 2006). It consists of RNA-Seq expression profiles for 87 lung adenocarcinoma and 77 adjacent normal lung tissues. From now on, we will refer to this data as LAUD_E. We used the LAUD data from TCGA as the training data and LAUD_E as the test data.

miRcorrNet was applied on mRNA and miRNA expression profiles of LUAD, which produced a list of significant genes. This gene list was used to train LAUD and test the performance of the tool using the external data, LAUD_E. In these experiments, we have considered top 30, top 5, top 2, and top 1 genes. The results are shown in the last 4 rows of Table 11. We observed that the generated classifier model resulted in high accuracy. The top 5 genes identified for LUAD are PLAC9, C2ORF71, FMO2, S1PR1 and AOC3. Moreover, we performed an additional test via selecting random lists of genes (30, 5, 2, and 1 genes). We have repeated this randomization experiment five times and calculated the mean. The results are presented on the rows of Table 11 with a title of LAUD (as the training data) and tested on LAUD_E (as the testing data) with the corresponding number of random genes (rows 5-8). Comparing results of random selected genes with the significant genes (rows 5-8 and rows 9-12) we see that for the significant genes, the results are significantly higher. Those observations prove that the list of significant genes suggested by our tool is more robust.

**Table 11:** Performance results obtained by applying different experiments on the validation data.

Experiments	Sensitivity	Specificity	Accuracy
LAUD_E random 1	0.63	0.52	0.59
LAUD_E random 2	0.58	0.71	0.63
LAUD_E random 5	0.73	0.77	0.74
LAUD_E random 30	0.92	0.98	0.94
LAUD (train) test on LAUD_E random 1	0.53	0.61	0.56
LAUD (train) test on LAUD_E random 2	0.53	0.73	0.62
LAUD (train) test on LAUD_E random 5	0.73	0.76	0.74
LAUD (train) test on LAUD_E random 30	0.87	0.94	0.91
LAUD (train) test on LAUD_E top 1	0.86	0.75	0.81
LAUD (train) test on LAUD_E top 2	0.76	0.97	0.86
LAUD (train) test on LAUD_E top 5	0.97	0.92	0.95
LAUD (train) test on LAUD_E top 30	0.98	0.97	0.97

## Discussions

### miRcorrNet prioritizes pan-cancer regulating miRNAs

**Figure 4:**
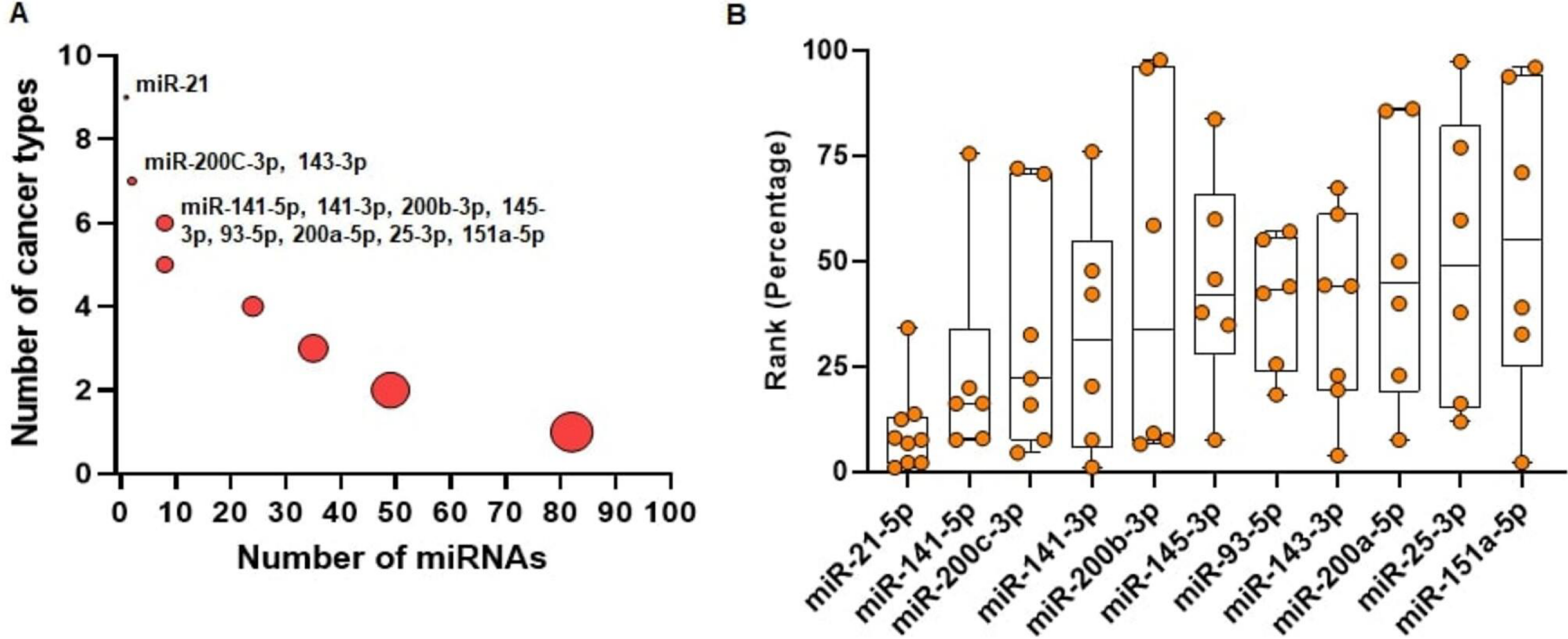
Pan-cancer regulating miRNAs predicted by miRcorrnet. (A) Eleven miRNAs the potentially regulate 6 or more cancer types, are highlighted. (B) Ranks of these 11 miRNAs in individual cancer types are denoted by dots. These miRNAs are sorted based on their median rank.

We run miRcorrNet to identify critical miRNAs in 11 TCGA cancer types. In miRBase database (Release 22.1), more than 2500 miRNAs are available (Kozomara, Birgaoanu & Griffiths-Jones, 2019). Among these miRNAs, miRcorrNet prioritized only a few (13 ~ 92, median = 43) miRNAs as critical for each specific cancer type. We also investigated how recurrently these miRNAs were prioritized across the cancer types. We determined that 11 miRNAs had recurrent gene regulation across 6 or more cancer types (as shown in Fig. 4). Among these miRNAs, miR-21-5p was associated with 9 cancer types, miR-200C and miR-143-3p were associated with 7 cancer types, and 8 other miRNAs had recurrent association with 6 cancer types (as shown in Fig. 4A). miR-21-5p was not only associated with the highest number of cancer types, but is also regarded as one of the top ranked miRNAs consistently across the cancer types (as shown in Fig. 4B). The rankings were based on the frequency score derived from the miRcorrNet algorithm. miR-21-5p is a well-known onco-miRNA whose elevated expression is linked with suppression of tumor suppressor genes associated with proliferation and apoptosis across numerous cancer types (Bandyopadhyay et al., 2010; Feng & Tsao, 2016). Moreover, diagnostic and prognostic roles of miR-21-5p and its implication in drug resistance had also been observed in many cancer types (Zhang et al., 2012; Faragalla et al., 2012; Wang et al., 2014; Yang et al., 2015; Feng & Tsao, 2016; Gaudelot et al., 2017; Emami et al., 2018). The literature-based evidence validated that miRcorrNet accurately predicted miR-21-5p as a critical pan-cancer regulator. The miRNAs miR-141-5p, miR-200C-3p, miR-141-3p, and miR-200b-3p were ranked as 2^nd^ to 5^th^, respectively (as shown in Fig. 4B). Both mature strands of miR-141 were prioritized as top critical miRNAs in our study, and concordant dysregulation of miR-141-5p and miR-141-3p across cancer types has been recently reported(Mitra, Sun & Zhao, 2015; Mitra et al., 2020). However, due to the historical belief that one mature strand is degraded during miRNA biogenesis, little is known about the coordinated regulatory roles of this 5p/3p pair. Here our study indicates that the miR-141 5p/3p pair mediates recurrent regulations across the cancer types, suggesting that they may be critical and selected during tumorigenesis. Among the 11 top ranked miRNAs, 5 of them (miR-141 5p, miR-200C-3p, miR-141-3p, miR-200B-3p, and miR-200a-5p) are the members of the miR-200 family. Interactions between the miR-200 family of miRNAs and ZEB1/ZEB2, two transcription factors that regulate epithelial to mesenchymal transition (EMT), inhibited EMT and suppressed cancer metastasis) (Korpal & Kang, 2008; Mongroo & Rustgi, 2010). The miRNAs miR-21-5p, miR-200C-3p, miR-143-3p, and miR-25-3p are among the 30 miRNAs that constitute, on average, 90% and 80% of all miRNA expression across the TCGA normal tissues and cancer tissues, respectively. Taken together, these results suggest that miRcorrNet is able to accurately prioritize pan-cancer regulating high-confidence miRNAs.

### Comparison with existing tools

There are a few web-based and R-based (Bunn & Korpela, 2021) tools to perform an integrated miRNA–mRNA analysis. anamiR is a R-based tool that integrates mRNA and miRNA profiles (Wang et al., 2019). The tool firstly determines DE mRNAs and miRNAs. Afterwards, it calculates the correlation scores for all possible DE mRNA and miRNA combinations. anamiR makes use of various miRNA-target prediction algorithms and validated databases. Lastly, anamiR performs functional analysis for the genes of interest. miRComb is another R based tool that conducts an integrated analysis of mRNA and miRNA expression (Vila-Casadesús, Gironella & Lozano, 2016). It first detects DE miRNAs and mRNAs. The mRNA–miRNA correlation values are then calculated, and negatively correlated mRNA–miRNA pairs are determined. Among these identified pairs, mRNA–miRNA interaction databases are used to detect the pairs that may be important for the disease. In this way, miRComb reports potentially important mRNA–miRNA pairs. In order to understand the biological functionality of these pairs, miRComb allows functional analysis. Compared to miRComb, anamiR allows the use of validated databases, while also allowing functional analysis. In addition to these R based tools there are also web-based tools. In this respect, MMIA tool uses the correlation information between miRNAs and mRNAs (Nam et al., 2009). It uses various target prediction algorithms to filter the predictions. MAGIA is another web based tool that is similar to MMIA (Sales et al., 2010). Different from MMIA, MAGIA offers 4 different methods for integrating miRNA and mRNA data. MirConnX is another such web based tool (Huang, Athanassiou & Benos, 2011). In addition to computed correlation values, mirConnX also uses biologically validated miRNA targets. Unfortunately, most of these web-based and R-based integrated miRNA and mRNA expression data analysis tools are not up to date, and they are discontinued. The availability status of these tools is presented in Table 12.

**Table 12:** List of correlation based tools for mRNA–miRNA integration.

Tool name	Data sets used	Link	Status
anamiR	Multiple Myeloma Prostate Cancer	R package	Not Available for R 4.0.4
miRComb	Colon Cancer Rectal Cancer Liver Cancer Stomach Cancer Esophageal Cancer	R package	Not Available for R 4.0.4
MMIA	ALL	http://cancer.informatics.indiana.edu/mmia (inactive)	Not Available
MAGIA	ALL	http://gencomp.bio.unipd.it/magia (inactive)	Not Available
MirConnX	GBM	http://www.benoslab.pitt.edu/mirconnx	Not Available
BCM	BRCA and THCA	http://doi.ieeecomputersociety.org/10.1109/TCBB.2015.2462370	Not Available

These similar studies such as anamiR and miRComb have some other limitations. Firstly, these studies use multiple target gene prediction algorithms. As a result of the use of target gene prediction algorithms, the number of identified target genes can be up to 4,000. It is not feasible to validate such a huge number of target genes using low throughput methods such as luciferase reporter assays. On the other hand, miRcorrNet predicts on average 407 genes as target genes, which is very low compared to other studies. This would help the experimental biologists to pinpoint and verify most interesting targets and their functions. Secondly, two R based tools developed for this purpose (anamiR, miRComb) are not easy to use for experimental biologists. On the contrary, miRcorrNet is extremely user friendly, which could help the clinicians and experimental biologists to easily obtain targets of desired miRNAs. Lastly, the tool that we developed in this study, miRcorrNet, uses state-of-the-art machine learning techniques and it includes a ranking step to separate the two classes, namely case and control, which is not available in other tools. In summary, the merit of our tool is different from other existing tools that deal with miRNA and mRNA expressions.

The goal of miRcorrNet is not to compete with other machine learning based tools that perform feature selection and classification tasks. Although miRcorrNet has an equally high performance as other tools reported in literature, the intended usage of miRcorrNet is completely different. The objective of miRcorrNet is to detect significant miRNA groups that may be able to serve as a biomarker for the disease. These significant miRNA groups should be considered for further analysis in order to deepen our understanding of the role of miRNA in a specific disease. Traditional tools provide a list of significant genes that are not related to any biological background, which causes the researcher to evaluate those significant genes by using other tools for enrichment analysis. However, researchers need a small specific set of genes that can be investigated to determine their contribution to the initiation and/or progression of the disease of interest. Therefore, one can regard miRcorrNet as a tool that identifies significant genes that are linked with a specific miRNA, whereas traditional approaches search for significant genes that are able to distinguish between the two-classes, hoping that using pathway analysis will shed light on those genes.

Although miRcorrNet provides ease of use, it has one shortcoming. In its current form, miRcorrNet performs the rank process only by using the relevant group; but intergroup relationships are not evaluated. We expect that more accurate results can be obtained when the combinations of groups are taken into account. We would like to implement this idea as a future work.

## Conclusion

Exploring the potential biological function of differential expressed genes through integrating multiple -omics data including miRNA and mRNA expression profiles, is a popular research topic. Nevertheless, how to assess the repression effect on target genes via integrating miRNA and mRNA expression profiles are not fully resolved. In this study, we proposed a novel tool, miRcorrNet, which conducts machine learning-based integration of expression profiles. The tool integrates miRNA and mRNA expression profiles in order to detect miRNA-associated genes that are able to perform the classification task. The tool detects groups, which are later subject to the Rank procedure. The groups consist of a set of genes that are associated with a specific miRNA. The strength of miRcorrNet is that the identified set of genes, that are represented in groups are guaranteed to distinguish two classes (cases vs. controls). Thus, those groups of genes and their associated miRNAs may serve as a biomarker for the specific disease under investigation.

## Additional Information and Declarations

### Data Availability

The following information was supplied regarding data availability: The miRcorrNet tool is available at GitHub: https://github.com/malikyousef/miRcorrNet. The data is available at TCGA (Project ID, dbGaP Study Accession, Project Name): TCGA - BLCA, phs000178, Bladder Urothelial Carcinoma; TCGA - BRCA, phs000178, Breast Invasive Carcinomal TCGA - KICH, phs000178, Kidney Chromophobe; TCGA - KIRC, phs000178, Kidney Renal Clear Cell Carcinoma; TCGA - KIRP, phs000178, Kidney Renal Papillary Cell Carcinoma; TCGA - LUAD, phs000178, Lung Adenocarcinoma; TCGA - LUSC, phs000178 Lung Squamous Cell Carcinoma; TCGA - PRAD, phs000178, Prostate Adenocarcinoma; TCGA - STAD, phs000178, Stomach Adenocarcinoma; TCGA - THCA, phs000178, Thyroid Carcinoma; TCGA - UCEC, phs000178, Uterine Corpus Endometrial Carcinoma
